# The optimal dietary crude protein level improves goat production performance by enhancing the body’s antioxidant function and energy metabolism

**DOI:** 10.3389/fmicb.2026.1734810

**Published:** 2026-02-06

**Authors:** Chunhui Wang, Caixia Zhang, Ali Mujtaba Shah, Zhisheng Wang, Shixiu Qiu, Zhenying Xu, Bai Xue, Lizhi Wang, Rui Hu, Huawei Zou, Yahui Jiang, Jianxin Xiao, Quanhui Peng

**Affiliations:** 1 Institute of Animal Nutrition, Key Laboratory of Bovine Low-Carbon Farming and Safety Production, Sichuan Agricultural University, Chengdu, China; 2 Chengdu Academy of Agriculture and Forestry Sciences, Chengdu, China

**Keywords:** antioxidant function, Chuannan black goat, metabolomics, metagenomics, protein levels

## Abstract

The current research was conducted to evaluate the impact of various crude protein (CP) concentrations in diets on growth performance, nutrient digestibility, nitrogen deposition, rumen fermentation, microbial community, and serum metabolomics in growing goats. Fifty healthy 4-month-old Chuannan black goats (*Capra hircus*) with similar body weight (13.75 ± 0.27 kg) were randomly distributed into 5 groups. Goats were fed diets with five different levels of CP: 8.12% (T8), 10.15% (T10), 12.17% (T12), 14.13% (T14), and 16.18% (T16), respectively. The total duration of the trial was 70 d, including a 14-day adaptation period. The average daily gain and feed conversion ratio displayed a quadratic upsurge and reduce respectively, with the rise of CP content in the diet. The group T14 exhibited the highest average daily gain and demonstrated the best feed conversion efficiency. A linearly (*p* < 0.05) increase of the digestibility of dry matter, neutral detergent fiber, and acid detergent fiber was observed, whereas a quadratic effect (*p* < 0.001) on nitrogen intake, fecal nitrogen, and urinary nitrogen was obtained with the increase of dietary CP. Moreover, dietary CP levels had a quadratic effect on the concentration of ruminal ammonia nitrogen (*p* = 0.021), rumen microbial protein (*p* = 0.042), total volatile fatty acid (*p* = 0.012), acetate (*p* = 0.040), isobutyrate (*p* = 0.024), and isovalerate (*p* < 0.001). Microbial metagenomics results showed that the relative abundance of *Burkholderia* and *Bacillus* increased (*p* < 0.05), while the relative abundance of *Pseudomonas* and *Salmonella* decreased (*p* < 0.05) when comparing group T14 to group T8. Metabolomic results showed that differently expressed metabolites were found to enrich the proline, glutathione and arginine metabolism, and citric acid cycle metabolic pathway. The concentration of serum genistein was positively correlated (*p* < 0.05, r = 0.665) with the abundance of *Bacillus* and negatively correlated (*p* < 0.05, r = −0.734) with the abundance of *Pseudomonas*. It is concluded that a dietary CP level of 14% enhances the antioxidant function and energy metabolism of the goats by altering the composition of rumen microorganisms, thereby improving production performance.

## 1 Introduction

The Chuannan black goat (*Capra hircus*) is an exceptional local goat breed in Southwest China and is also highly regarded as a commercial mutton goat breed in the country. It possesses favorable traits such as strong adaptability, tolerance to rough feed, rapid growth and development, high reproduction rate, and delicious meat. Dietary protein is crucial for animals as it participates in numerous metabolic processes and plays a vital role in the immune system (Yang et al., 2022; Van Soest et al., 1991; Zhao et al., 2020). Lima Júnior et al. (2023) reported that the net protein (NP) and metabolizable protein (MP) requirements for Caninde goats were 2.19 and 1.88 g/kg EBW^0.75^ (Empty Body Weight^0.75^), respectively. However, Almeida et al. (2020) claimed that NPg did not vary among goat genotypes (meat, dairy, and indigenous). Elhadi et al. (2025) used 48 Lacaune dairy ewes in early lactation, they found that higher dietary protein (16.6% CP) maintained milk yield but increased milk urea (+18%) while reducing milk fat (−4%) and solids (−2%), and elevated plasma urea (+45%). Lower protein (14.8% CP) decreased plasma urea (−45%) and increased glucose (+6%). Noviadi et al. (2022) assessed the effects of six protein levels (12–17%) in cassava leaf complete silage on the production performance of 24 local goats, with results showing that the 17% protein level significantly increased feed intake and daily weight gain, while the 14% protein level achieved the optimal feed conversion ratio. Results showed that although excessive protein supplementation increased weight gain, it elevated metabolic burden and led to a decline in feed utilization efficiency. Although we all know that an appropriate level of dietary protein can promote goat growth, the specific underlying mechanism remains unclear.

Microbial metagenomics holds great potential and offers unique advantages in exploring the microbial world and understanding their interactions with the environment and hosts. Earlier research displayed that the composition and activity of rumen microbial communities are influenced by various factors, including diet and gender (Wang et al., 2022). Compared with goats fed a diet containing 13.0% CP, the relative abundance of Bacteroidetes and Fibrobacteres increased, while the relative abundance of Proteobacteria and Synergistetes reduced in the rumen of goats fed diet with 10.0% CP (Zhang et al., 2020). Fu et al. (2025) fed 80 Yunshang Black goats with four dietary regimens to investigate their effects on cecal fermentation, immunity, and microbiota. They found that the high-energy high-protein (HEHP) combination significantly increased cecal microbial diversity (Shannon/Simpson indices) and valerate production while elevating Firmicutes phylum abundance (e.g., *Clostridium* genus). In other words, the composition of rumen microorganisms is closely related to the level of dietary protein content. Metabolomics, as a branch of omics concerned with the comprehensive analysis of small molecule metabolites within biological systems, exhibits distinct characteristics and advantages (Gertsman and Barshop, 2018). Metabolites are downstream products of cellular processes and are thus highly sensitive indicators of nutrition variations, environmental exposures, and disease states. This sensitivity makes metabolomics a powerful tool for understanding complex biological mechanisms (Levy et al., 2017; Wang et al., 2021). Liu et al. (2025) selected eight healthy Leizhou goats weighing 11 ± 0.78 kg and raised them on lower-protein diets with varying energy levels, aiming to evaluate the effects of consuming lower-protein diets with different energy levels on the fecal bacterial community and metabolomics in goats. Fecal metabolomic analysis revealed that following the intake of lower-protein diets with different energy levels, various differential metabolites were observed in the goats. Compared to the low-energy group, the other three groups showed enhanced lipid metabolism and amino acid metabolic pathways. Zhu et al. (2024) investigated the effects of supplementing resveratrol and *β*-hydroxy-β-methylbutyric acid at different dietary protein levels on the rumen microbial and metabolite composition of Tibetan sheep. The results revealed that differential metabolites were primarily enriched in metabolic pathways such as pyrimidine metabolism, glycine/serine/threonine metabolism, and ATP-binding Cassette transporters. The supplementation of resveratrol and β-hydroxy-β-methylbutyric acid in the 14% crude protein diet effectively improved ruminal digestive enzyme activity and antioxidant capacity by modulating the microbial community and regulating metabolic activities. However, to date, there has been limited research using metagenomics and metabolomics collaboratively to elucidate the mechanism by which an appropriate protein level enhances goat production performance.

Therefore, the purpose of this research was to figure out why an appropriate dietary level can promote Chuannan black goat growth from the perspectives of microorganisms and metabolomics, through growth, digestion, and metabolism experiments. We hypothesized that an appropriate level of dietary protein can alter the composition of rumen microorganisms, thereby changing the antioxidant and anti-inflammatory capabilities of goat.

## 2 Materials and methods

### 2.1 Animal ethics statement

All the required permissions regarding Animal care and management for the current research were received from the Institutional Animal Care and Sichuan Agricultural University committee (Ethics No. SYXK (chuan) 2019–187).

### 2.2 Animal experiment design and management

Fifty 4-month-old Chuannan black goats (*Capra hircus*) with similar body weight (13.75 ± 0.27 kg) were selected from Jintang County, Chengdu, China. The goats were numbered with ear tags and randomly divided into 5 groups with 10 replicates per group, and animals in each group were housed in individual cages. The study included 5 diets with CP levels of 8.12% (T8), 10.15% (T10), 12.17% (T12), 14.13% (T14), and 16.18% (T16), respectively, corresponding to 66.7, 83.3, 100, 117.7, and 133.3% of the recommended protein level based on the weight of 15 kg and average daily gain (ADG) 150 g specified in the Chinese Feeding Standard of Meat-type Sheep and Goat (NY/T 816–2021). The composition and nutritional value of diet was presented in Table 1. A 14-day adaptation period was provided to goats before the research trial, and the research trial lasted 56 days. During the entire study, a total mixed ration was provided to all goats twice daily on 8:00 and 16:00. The leftover in the trough was collected and weighed before feeding the next morning, and recorded as the leftover feed weight. The feed provided in the morning was maintained about 5% feed refusal.

**Table 1:** Diets composition and nutritional levels (%, dry matter basis).

Ingredients	Dietary protein level				
	T8	T10	T12	T14	T16
Corn	41.00	34.00	27.00	19.00	12.00
Wheat bran	6.00	5.50	3.50	2.50	2.00
Soybean meal	4.00	6.50	9.00	11.50	13.00
Rapeseed meal	0.50	0.80	1.50	2.00	3.00
Distillers dried grains with solubles	2.00	4.00	6.00	8.00	11.00
Alfalfa hay	4.00	13.00	22.50	31.30	40.00
Wheat straw	30.00	27.00	22.98	19.00	14.00
Corn silage	8.20	5.30	4.00	3.45	2.00
Calcium carbonate	1.19	1.00	0.75	0.60	0.40
Dicalcium phosphate	0.61	0.40	0.27	0.15	0.10
Sodium bicarbonate	0.50	0.50	0.50	0.50	0.50
Sodium chloride	1.00	1.00	1.00	1.00	1.00
Premix^1^	1.00	1.00	1.00	1.00	1.00
Total	100.00	100.00	100.00	100.00	100.00
Nutrient level^2^					
DM, %	93.10	93.46	93.55	93.84	94.14
ME, MJ/kg DM	8.41	8.43	8.44	8.47	8.52
CP, %	8.12	10.15	12.17	14.13	16.18
NDF, %	37.00	38.66	42.30	43.09	43.99
ADF, %	21.30	21.61	25.54	26.70	28.40
Ca, %	0.80	0.82	0.83	0.84	0.86
P, %	0.45	0.46	0.47	0.50	0.50

DM, dry matter; ME, metabolizable energy; CP, crude protein; NDF, neutral detergent fiber; ADF, acid detergent fiber.

1 The premix provides per kilogram of diet: vitamin A, 6000 IU, vitamin E, 40 mg, vitamin D_3_, 1,500 IU, Cu (as copper sulfate) 10 mg, Fe (as ferrous sulfate) 80 mg, Mn (as manganese sulfate) 50 mg, Zn (as zinc sulfate) 60 mg, I (as potassium iodide) 0.5 mg, Se (as sodium selenite) 0.3 mg, Co (as cobalt chloride) 0.2 mg.

2 ME was calculated values and the rest nutrients were measured values. ME was calculated according to nutrient requirements of small ruminants.

### 2.3 Collections of the samples and analysis

#### 2.3.1 Chemical analysis

All feces, diets, and refused samples collected during the experiment were dried at 65 °C for 48 h and ground to pass through a 0.9-mm screen before analysis. The association of official analytical chemist (AOAC 1990) method was used to determine the dry matter (DM), crude protein (CP), calcium, and phosphorus in the samples. Meanwhile, the protocol of Van Soest et al. (1991) was followed to determine the acid detergent fiber (ADF) and neutral detergent fiber (NDF).

#### 2.3.2 Growth performance

On the morning of day 0 and day 56 of the trial period, before morning feeding body weight of all goats were measured. The weights were recorded as initial body weight (IBW) and final body weight (FBW) respectively, and the ADG was calculated. The weight of feed offered to the goats was measured each day, and the leftovers were collected daily to calculate the average feed intake (ADFI). The feed conversion ratio is calculated by dividing the ADFI by the ADG.

#### 2.3.3 Digestion and metabolism experiment

Six goats with similar body weights in each group were chosen and placed into metabolic cages measuring 1,600 mm × 750 mm × 1,200 mm. The ADFI, fecal volume, and urine volume of each goat were recorded for 4 consecutive days. Approximately 10% of the fecal samples were retained, and 10% sulfuric acid was added to avoid nitrogen loss before freezing under −20 °C for future determination. Plastic basin with 10% of sulfuric acid was used for collection of the urine samples. The total urine volume was measured and recorded with a measuring cylinder. Then, 10% of the total urine volume was sampled and frozen at −20 °C for future nitrogen measurement.

#### 2.3.4 Rumen fermentation and metagenomics

Rumen fluid was collected via an oral gastric tube 4 h post-feeding on day 56. The pH was measured immediately. Concentrations of ammonia nitrogen (NH3-N), microbial protein (MCP), and volatile fatty acids (VFAs) were determined as described by Ma et al. (2021). For microbial analysis, genomic DNA was extracted from T8 and T14 samples, and PE150 sequencing was performed on the NovaSeq platform. Taxonomic and functional profiling were conducted using Kraken 2 and the KEGG database.

#### 2.3.5 Serum metabolomics

Serum was separated from jugular vein blood collected on day 56 and stored at −20 °C. Samples were analyzed using LC–MS (Waters, USA) with a Q Exactive mass spectrometer (Thermo Fisher Scientific, USA). Data processing and multivariate modeling (PCoA and OPLS-DA) were performed using R software. Differential metabolites were identified based on *p* < 0.05 and VIP > 1.

### 2.4 Statistical analysis

A randomized complete design was used to determine the effect of protein level on various parameters using the Mixed procedure with SAS version 9.4 (SAS Institute Inc., Cary, NC). The model used to analyze these data was:


$$Y_{\mathrm{ij}}=\mu +\mathrm{Pro}t_{i}+E_{\mathrm{ij}}$$

where *μ* = the general mean, *Prot_i_* = the effect of the protein level *i*, and *E_ij_* = the random error. Means were separated using orthogonal polynomial contrasts to examine linear or quadratic effects of protein level after protein level effect was detected significant. Data normality test was performed using the Shapiro–Wilk procedure and significance was declared at *p* < 0.05, while a trend was regarded at 0.05 ≤ *p* < 0.10. The Spearman correlation analysis was conducted on the 35 selected metabolites and the top four microbial genera with significant differences using Origin software (version 9.8.0.200).

## 3 Results

### 3.1 Growth performance

Table 2 presented the results of the growth performance. The protein content in the diet had a quadratic impact on FBW (*p* < 0.05), ADG (*p* < 0.001) and FCR (*p* < 0.001). An increasing trend in ADFI was observed with the rise of dietary CP content (Not Significant, NS). The ADG (*p* < 0.001) and FCR (*p* < 0.05) of group T14 was the best compared to other groups.

**Table 2:** Impact of various concentrations of protein in diet on growth performance in growing goat.

Item	Treatments					SEM	*p*-value	Contrast	
	T8	T10	T12	T14	T16			L	Q
IBW(kg)	13.95	13.86	13.70	13.84	13.93	0.273	0.942	0.976	0.961
FBW(kg)	16.93^b^	18.54^ab^	18.99^ab^	21.39^a^	19.51^ab^	0.449	0.028	0.010	0.014
ADFI(kg/d)	0.69	0.76	0.77	0.82	0.69	0.018	0.109	0.711	0.075
ADG(kg/d)	0.05^c^	0.08^b^	0.09^b^	0.14^a^	0.10^b^	0.005	<0.001	<0.001	<0.001
FCR	13.80^a^	9.51^b^	8.54^b^	5.86^b^	6.90^b^	0.417	0.001	<0.001	<0.001

IBW, initial body weight; FBW, final body weight; ADFI, average daily feed intake; ADG, average daily gain; FCR, feed conversion ratio; L, linear; Q, quadratic.

a, b, c Means within rows with different superscripts differ *p* < 0.05.

### 3.2 Apparent digestibility of nutrients and nitrogen metabolism

Table 3 presented the results of the apparent digestibility of nutrients and nitrogen metabolism. A linear effect was noticed in the apparent digestibility of ADF (*p* < 0.05), while quadratic effects were observed in the apparent digestibility of DM (*p* < 0.05) and NDF (*p* < 0.05). A quadratic trend in the digestibility of NDF was observed as the dietary CP level increase (NS).

**Table 3:** Impact of various concentrations of protein in diet on nutrients apparent digestibility and nitrogen metabolism in growing goats.

Item	Treatments					SEM	*p-*value	Contrast	
	T8	T10	T12	T14	T16			L	Q
Nutrients digestibility									
DM(%)	62.88^b^	63.68^b^	65.51^a^	66.95^a^	57.03^b^	1.612	0.005	0.010	0.041
CP(%)	60.01^b^	62.86^b^	68.91^a^	72.73^a^	55.52^b^	1.994	0.002	0.069	0.063
NDF(%)	47.90^a^	48.13^a^	48.82^a^	50.40^a^	36.00^b^	2.370	0.010	0.009	0.038
ADF(%)	35.00^b^	35.51^b^	42.38^a^	45.64^a^	30.68^b^	2.687	0.011	0.037	0.120
Nitrogen metabolism									
NI(g/d)	8.69^e^	10.67^d^	14.03^c^	16.68^b^	17.99^a^	0.946	<0.001	<0.001	<0.001
FN(g/d)	2.48^c^	3.51^b^	3.52^b^	3.54^b^	6.33^a^	0.366	<0.001	<0.001	<0.001
UN(g/d)	3.92^b^	4.22^ab^	4.57^ab^	5.04^a^	5.29^a^	0.202	<0.001	<0.001	<0.001
NR(g/d)	2.29^b^	2.94^b^	6.10^a^	8.10^a^	6.37^a^	0.644	<0.001	<0.001	0.001
NR/NI(%)	26.36^d^	27.56^c^	43.47^a^	48.56^a^	37.41^b^	0.030	0.012	0.042	0.034

DM, dry matter; CP, crude protein; NDF, neutral detergent fiber; ADF, acid detergent fiber; NI, nitrogen intake; FN, fecal nitrogen; UN, urinary nitrogen; NR, nitrogen retention; L, linear; Q, quadratic.

a, b, c, d, e Means within rows with different superscripts differ *p* < 0.05.

The dietary crude protein content had quadratic effect on nitrogen intake (NI) (*p* < 0.001), fecal nitrogen (FN) (*p* < 0.001), urinary nitrogen (UN) (*p* < 0.001), nitrogen retention (NR) (*p* = 0.001) and NR/NI (*p* < 0.05) (Table 3).

### 3.3 Parameters of rumen fermentation

The results of the rumen fermentation parameters are presented in Table 4. A quadratic effect was observed in NH_3_-N (*p* < 0.05), MCP (*p* < 0.05), TVFA (*p* < 0.01), acetate (*p* < 0.05), valerate (*p* < 0.01) and isovalerate (*p* < 0.001) with the increase in dietary protein content. Nevertheless, no substantial variation was noted in the ruminal pH value, propionate and butyrate, and the A: P.

**Table 4:** Impact of various concentrations of protein in diet on rumen fermentation parameters in growing goats.

Item	Treatments					SEM	*p*-value	Contrast	
	T8	T10	T12	T14	T16			L	Q
pH	6.75	6.76	6.78	6.79	6.44	0.619	0.246	0.053	0.072
NH_3_-N(mg/dL)	11.97^b^	18.62^b^	19.24^b^	26.65^a^	27.51^a^	1.184	0.021	0.014	0.025
MCP(mg/L)	3.43^b^	3.51^b^	3.56^b^	4.69^a^	4.04^a^	0.207	0.042	0.016	0.013
TVFA(mmol/L)	61.60^c^	67.18^c^	71.12^b^	78.48^a^	72.07^b^	3.645	0.012	0.010	0.006
Acetate(mmol/L)	43.50^c^	47.28^bc^	49.97^bc^	54.40^a^	50.36^b^	1.886	0.040	0.069	0.014
Propionate(mmol/L)	14.20	15.45	16.27	17.60	16.35	0.648	0.272	0.601	0.609
Isobutyrate(mmol/L)	0.32^b^	0.36^b^	0.43^b^	0.66^a^	0.59^a^	0.052	0.024	0.007	0.026
Butyrate(mmol/L)	3.06	3.45	3.74	4.50	4.00	0.203	0.110	0.036	0.110
Isovalerate(mmol/L)	0.25^b^	0.26^b^	0.29^b^	0.66^a^	0.30^b^	0.037	<0.001	<0.001	<0.001
Total BFCA(mmol/L)	0.77^bc^	0.61^c^	0.60^c^	0.96^b^	1.32^a^	0.113	<0.001	<0.001	<0.001
Valerate(mmol/L)	0.26^c^	0.36^c^	0.40^b^	0.62^a^	0.45^b^	0.040	0.012	0.032	0.002
A:P	3.03	3.06	3.07	3.09	3.08	0.084	0.213	0.237	0.482

NH_3_-N, ammonia nitrogen; MCP, microbial protein; TVFA, total volatile fatty acid concentration; A:P, Acetate:Propionate ratio; L, linear; Q, quadratic.

a, b, c Means within rows with different superscripts differ *p* < 0.05.

### 3.4 Metagenomics analysis of rumen microbial composition

**Figure 1:**
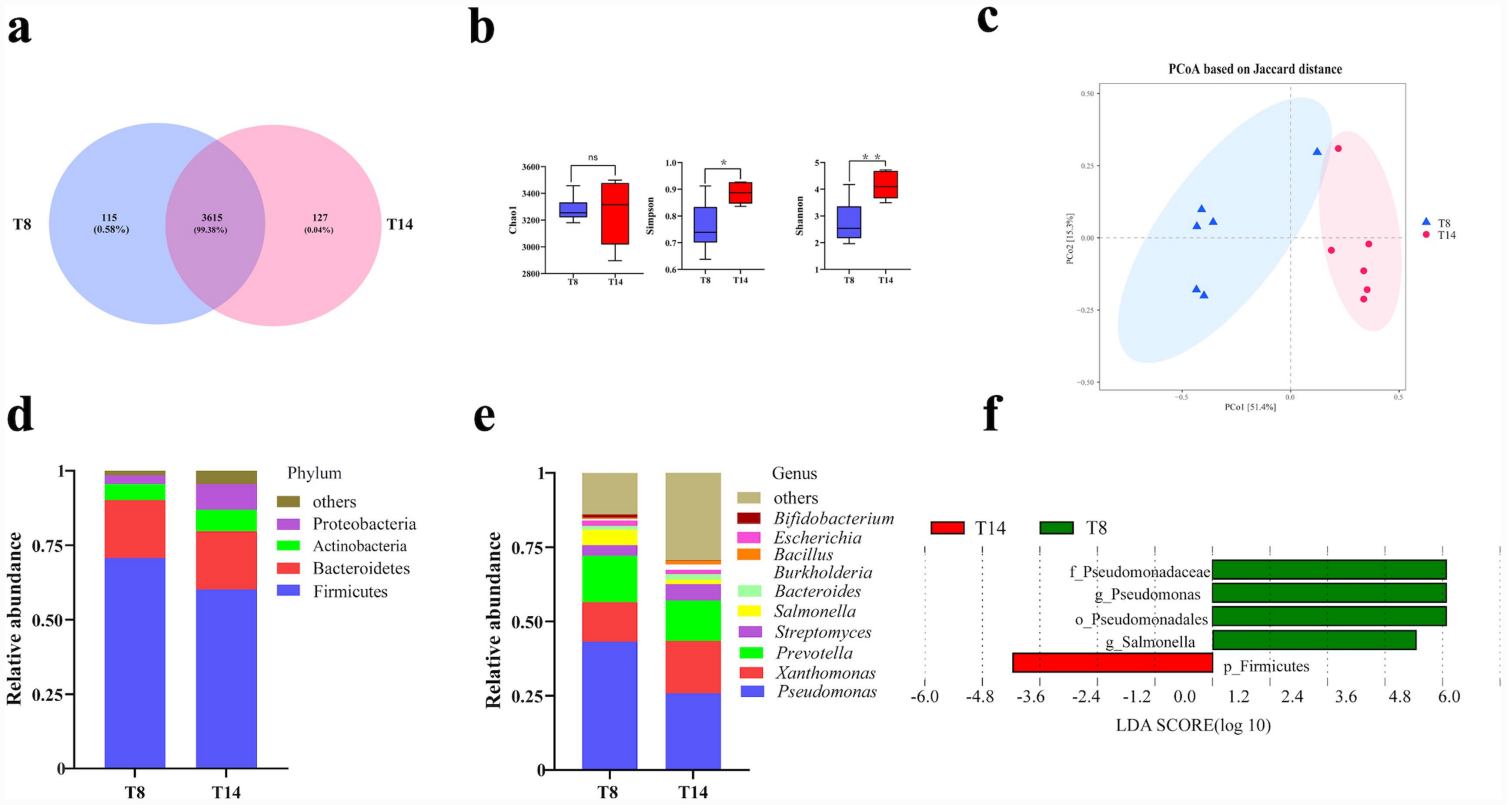
The comparative analysis of the microbial communities in the rumen of goats from groups T8 and T14. **(a)** Displays the Venn diagram of operational taxonomic units (OTUs), while **(b)** illustrates the alpha diversity indices (Chao1, Simpson, and Shannon) for the two groups. **(c)** Depicts the principal coordinate analysis (PCoA) of the microbial communities. **(d,e)** Provide the composition of the goat rumen microbiota at the phylum and genus levels, respectively. Lastly, **(f)** showcases the results of the linear discriminant analysis effect size (LEfSe) analysis, highlighting the differential ruminal microorganisms in groups T8 and T14. T8 denoted a dietary crude protein level of 8%, while T14 indicated a dietary crude protein level of 14%.

Since the production performance of the T14 and T8 groups was the best and the worst respectively, six heads from each of these 2 groups were selected for rumen microorganisms metagenomic analysis and serum metabolomic analysis. Utilizing the Illumina sequencing platform and following quality control procedures, a total of 195,150,361 and 192,696,573 high-throughput sequences were acquired from rumen fluid samples of groups T8 and T14. The percentage of sequences with an average quality score exceeding Q30 was 93%. A Venn diagram was constructed to illustrate the intersections between the two groups (Figure 1a), it revealed that 127 OTUs (0.04% of the total sequence) were exclusive to group T14, and 115 OTUs (0.58% of the total sequence) were unique to group T8, and 3,615 OTUs (99.38% of the total sequence) were shared between the two groups.

The alpha diversity indices of microbial taxa are presented in Figure 1b. There was no significant difference in ACE and Chao indexes between the two groups (NS). However, the Simpson and Shannon indexes (*p* < 0.05) of group T14 were higher than those of group T8. The beta diversity result showed distinguishable microbial communities between groups T8 and T14 (Figure 1c), with PCo1 and PCo2 explaining 51.4 and 15.3% of the total variance, respectively.

As depicted in Figure 1d and Table 5, the predominant bacteria in groups T8 and T14 at the phylum level were Firmicutes, Bacteroides, Actinobacteria, and Proteobacteria, collectively constituting over 95% of the total bacterial sequence. The relative abundance of Proteobacteria in group T14 was higher than that in group T8 (*p* < 0.05), whereas the relative abundance of Firmicutes in group T14 was lower than that in group T8 (*p* < 0.05). At the genus level, the top 10 taxa were depicted in Figure 1e and Table 5. The relative abundance of *Pseudomonas* and *Salmonella* was higher (*p* < 0.05), whereas the relative abundance of *Burkholderia* and *Bacillus* was lower (*p* < 0.05) in group T8 compared to group T14. The LefSe analysis yielded comparable results, as shown in Figure 1f. To gain a deeper insight into the functional alterations of rumen microbiota between group T14 and group T8, Kyoto Encyclopedia of Genes and Genomes enrichment analysis was conducted. At the level 3 (Table 6), it was observed that pathways related to amino acid metabolism, including alanine, aspartate, and glutamate metabolism, glycine, serine, and threonine metabolism, cysteine and methionine metabolism, arginine and proline metabolism, and histidine metabolism, were down regulated in group T14 compared to group T8.

**Table 5:** The composition and relative abundance of rumen bacterial communities in groups T8 and T14 analyzed at the phylum and genus level (%).

Item	T8	T14	SEM	*p*-value
Phylum level				
Firmicutes	70.70	60.12	0.150	0.241
Bacteroidetes	19.48	19.58	0.106	0.987
Actinobacteria	5.39	7.22	0.031	0.330
Proteobacteria	2.90	8.59	0.401	0.007
Others	1.54	4.50	0.182	0.010
Genus level				
*Pseudomonas*	43.19	25.82	0.136	0.018
*Xanthomonas*	13.31	17.64	0.081	0.385
*Prevotella*	15.61	13.55	0.089	0.709
*Streptomyces*	3.52	5.49	0.028	0.249
*Salmonella*	5.19	1.57	0.030	0.034
*Bacteroides*	1.34	1.89	0.009	0.336
*Escherichia*	1.78	1.42	0.010	0.568
*Burkholderia*	0.54	1.77	0.010	0.030
*Bacillus*	0.44	1.32	0.007	0.035
*Bifidobacterium*	1.18	0.16	0.012	0.154
Others	13.90	29.38	0.107	0.005

**Table 6:** Predicted functions at level 3 of the rumen bacterial microbiota of goats in groups T8 and T14.

Name	KO	EC	T8	T14	SEM	*p*-value
^1^Amino acid metabolism	K00812	EC2.6.1.1	3.57	3.14	2.681	0.009
^2^Alanine, aspartate and glutamate metabolism (5)	K00817	EC2.6.1.9	3.46	3.00	2.681	0.004
Glycine, serine and threonine metabolism (5)	K01928	EC6.3.2.13	2.86	2.45	2.49	0.009
Cysteine and methionine metabolism (5)	K00262	EC1.4.1.4	3.25	2.83	2.471	0.009
Valine, leucine and isoleucine degradation (3)	K01915	EC6.3.1.2	3.22	2.89	2.356	0.026
Valine, leucine and isoleucine biosynthesis (1)	K00928	EC2.7.2.4	3.12	2.76	2.174	0.041
Lysine biosynthesis (4)	K12524	EC2.7.2.4	3.10	2.75	2.279	0.026
Lysine degradation (1)	K01696	EC4.2.1.20	3.10	2.68	2.177	0.004
Arginine biosynthesis (4)	K00382	EC1.8.1.4	3.07	2.56	2.205	0.009
Arginine and proline metabolism (2)	K00133	EC1.2.1.11	3.04	2.65	2.202	0.015
Histidine metabolism (1)	K01955	EC6.3.5.5	3.00	2.69	2.037	0.041
Tyrosine metabolism (2)	K00826	EC2.6.1.42	2.93	2.58	2.045	0.132
Phenylalanine metabolism (2)	K00800	EC2.5.1.19	2.92	2.54	2.021	0.026
Tryptophan metabolism (1)	K00266	EC1.4.1.13	2.86	2.48	2.050	0.009
Phenylalanine, tyrosine and tryptophan biosynthesis (4)	K00491	EC1.14.14.47	2.88	2.54	2.048	0.065
	K01847	EC5.4.99.2	2.88	2.48	2.051	0.069
^1^Metabolism of other amino acids	K13788	EC2.3.1.8	2.88	2.42	2.054	0.078
Taurine and hypotaurine metabolism (1)	K01924	EC6.3.2.8	2.86	2.50	2.050	0.061
Cyanoamino acid metabolism (1)	K01921	EC6.3.2.4	2.89	2.51	2.049	0.032
D-Amino acid metabolism (1)	K05349	EC3.2.1.21	3.38	2.92	2.117	0.014

1 Representing the function of KO number comparison in KEGG at level 3.

2 Representing the number of genes involved in the pathway.

### 3.5 Metabolomic analysis of serum

**Figure 2:**
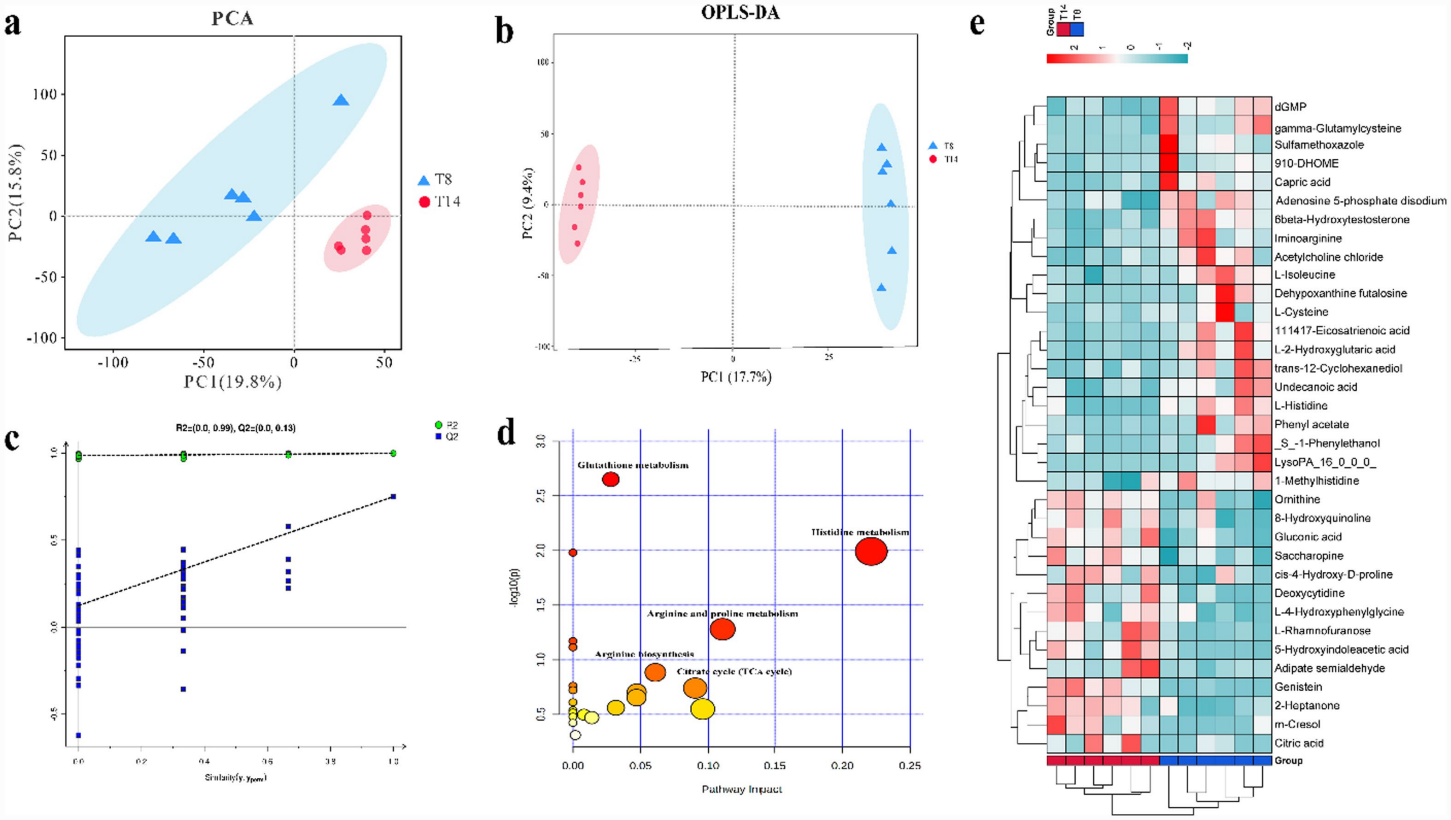
The comparative analysis of the serum metabolites of goats from groups T8 and T14. **(a)** Principal component analysis scores plot was generated to compare the serum metabolites of group T8 and T14 using LC/MS. The abscissa represents the score of the first principal component, while the ordinate represents the score of the second principal component, with different points indicating samples. **(b)** Shows the Orthogonal Partial Least Squares Discriminant Analysis (OPLS-DA) plot model comparing group T8 vs. T14. **(c)** Displays the permutations plot, while panel **(d)** presents the clustering heat map of significantly expressed metabolites between group T8 and T14. Lastly, **(e)** illustrates the metabolic pathway enrichment map when comparing group T14 to T8. The *x*-axis denotes the pathway impact, while the *y*-axis represents the value of −log (P). The larger sizes correspond to greater pathway enrichment, while the darker colors signify higher pathway impact values. T8 denoted a dietary crude protein level of 8%, while T14 indicated a dietary crude protein level of 14%.

The PCA score plots comparing group T8 and T14 was presented in Figure 2a. The T8 and T14 group were entirely distinguishable, with the first and second principal components explaining 19.8 and 15.8% of the overall variance, respectively. The OPLS-DA also showed a clear differentiation between the two groups (Figure 2b). The parameters of the model indicated high reliability and predictivity (R2X = 0.605, Q2 = 0.806). The permutation test indicated the absence of an over-fitting (Figure 2c).

**Figure 3:**
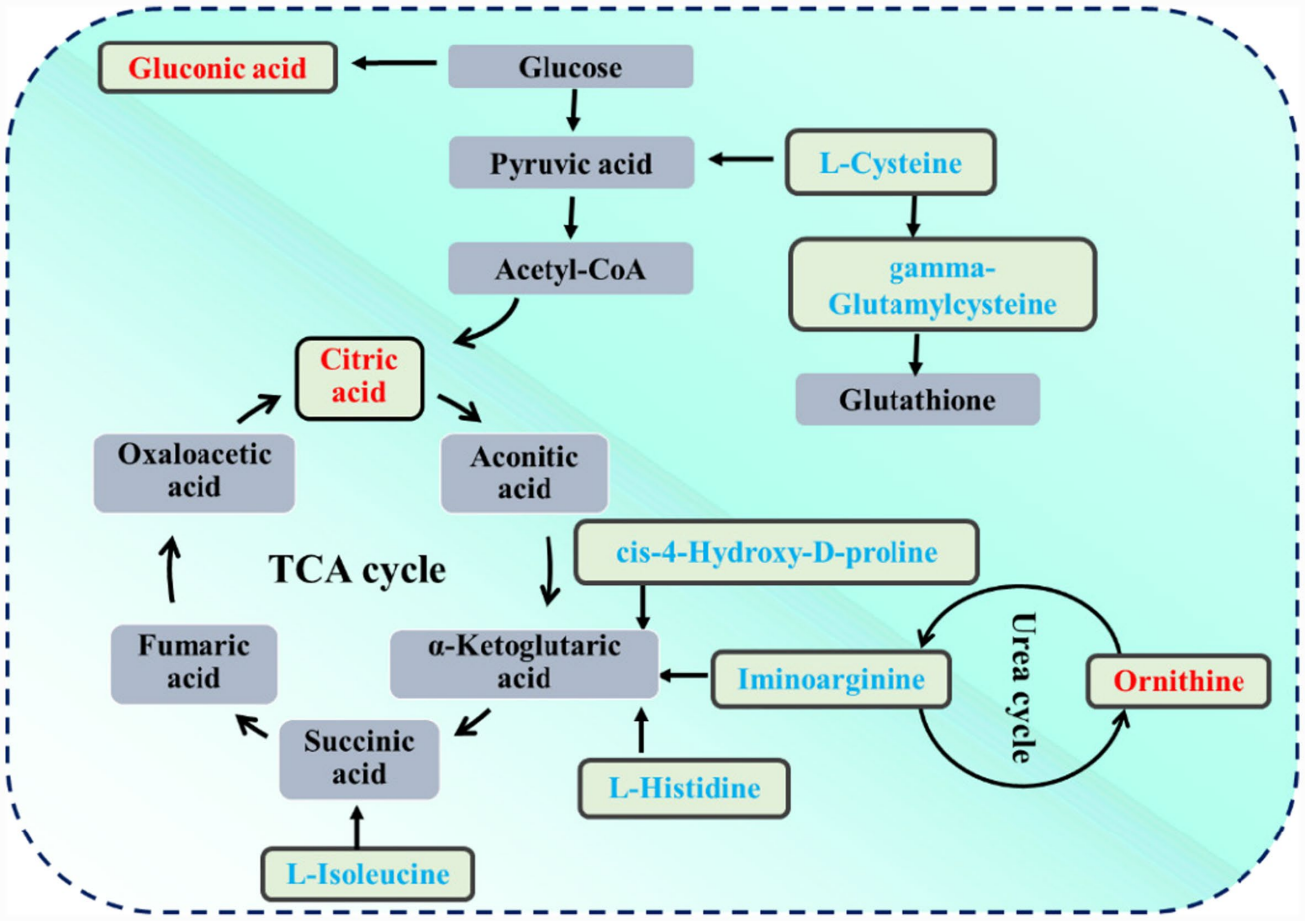
Summary diagram depicting the metabolic network comparison between group T14 and T8. The color red is used to denote metabolites that have been significantly upregulated, while blue is used to indicate metabolites that have been significantly downregulated. T8 denoted a dietary crude protein level of 8%, while T14 indicated a dietary crude protein level of 14%.

The identification of differently expressed metabolites between group T8 and T14 was conducted by utilizing the OPLS-DA model to obtain a VIP score >1, as well as with *p* values < 0.05. As depicted in Figure 2e and Supplementary Table S1, a total of 35 metabolites were found to be different. Among them, 14 metabolites such as genistein, 5-hydroxyindoleacetic acid, m-cresol, ornithine, and citric acid were upregulated, whereas 21 metabolites, including L-isoleucine, L-cysteine and L-histidine were downregulated. As depicted in Figure 2d and Supplementary Table S2, the differentially expressed metabolites were implicated in the pathway of histidine metabolism, arginine and proline metabolism, glutathione metabolism, and the citrate cycle. The schematic depiction of the metabolic network was illustrated in Figure 3.

## 4 Discussion

### 4.1 Growth performance

In the present study, the ADFI was not affected by the dietary CP levels, and similar results were reported before by Zhu et al. (2020). However, the ADG and FCR of the goats were significantly improved as the dietary protein level increased from 8 to 14%, which was consistent with previous studies. Titi et al. (2000) observed the highest ADFI and ADG in the group fed a 16% protein diet when Jordan black goat were provided with diets containing four different protein levels (12, 14, 16 and 18%). Wang et al. (2014) also reported that a 17% CP diet led to a higher ADG and an improved FCR compared to the 15% CP group of Hainan black goats. Our results showed that 14% CP was the optimal, while previous studies reported that 16 and 17% were the best. The difference is speculated to be due to the different breeds and ages of goats.

### 4.2 Apparent digestibility of nutrients and nitrogen metabolism

Our results showed that the apparent digestibility of DM, CP, NDF, and ADF was significantly affected by the dietary protein level. This finding was consistent with Zhu et al. (2020), who reported improved apparent digestibility of nutrients in Anhui white goats as dietary protein level increased. In this study, the apparent digestibility of NDF and ADF of group T14 was higher than that of group T8, this may be due to the higher amount of degradable protein entering the rumen, resulting in more nitrogen source available for the growth of microorganisms. Proper dietary protein content can meet the requirements of microbial growth better (Liu et al., 2021). With the increase of dietary protein content, the activity of fiber-degrading bacteria increased, leading to an increase in the apparent digestibility of NDF and ADF.

In the present study, the NI and FN increased with the increase of dietary protein level. Zhang et al. (2020) reported that the FN of goats increased by 20.9 and 36.45% when fed with 11.5 and 13.0% CP compared to the control group (CP, 10.0%). Additionally, as the dietary protein content increased from 10 to 16%, NR of goat kids increased from 3.6 to 5.6 g/day, respectively. Furthermore, the UN of goats increased with the increase of dietary protein content, which was in line with the results reported by Katongole et al. (2022), who observed in dairy heifers. These findings suggested that an appropriate dietary protein level is beneficial for enhancement of nitrogen retention in goats.

### 4.3 Parameters of rumen fermentation

Maintaining the rumen pH within the normal range is crucial for the health of rumiants, as any decrease or increase can disrupt the growth of rumen microbes. In this study, the dietary protein level did not have a significant effect on rumen pH, which remained within the normal range of 5.5–7.5 (Zhang et al., 2022). Putri et al. (2021) also reported that an elevation in dietary protein intake did not have significant impact on rumen pH levels. In the present study, the NH_3_-N concentration exhibited a linear increase in response to dietary protein levels, which was consistent with the findings of Aguerre et al. (2016), and elevated NH_3_-N concentrations were attributed to accelerated protein fermentation associated with higher rumen degradable protein content (Souza and White, 2021).

The current study demonstrated that the higher dietary protein led to elevated concentrations of TVFA in the rumen, which was consistent with the findings of Yang et al. (2016) observed in Chahaer lambs. In the present study, the concentration of propionic acid in group T14 was higher than that in group T8. As we all known that propionic acid serves as the primary substrate for gluconeogenesis in ruminants (Gleason et al., 2022). Consequently, it is suggested that the goat in group T14 exhibits greater energy utilization efficiency, potentially contributing to the superior growth performance. Zhou et al. (2019) and Zhang et al. (2022) documented that an increase in dietary protein level from 111 to 136 g/kg DM led to an elevated ruminal concentration of isobutyrate and isovalerate in beef cattle. The branched chain fatty acids in the rumen are primarily produced through breakdown of amino acids (Liu et al., 2023; Lu et al., 2024). The increase in dietary protein undoubtedly enhances the production of branched-chain fatty acids.

The observed shifts in rumen fermentation parameters are closely synchronized with alterations in the microbial community structure. In this study, the T14 group showed a higher abundance of beneficial bacteria such as *Bacillus* and *Burkholderia*, which are known to possess robust enzymatic capabilities for degrading complex polysaccharides. This microbial shift likely drives the observed quadratic increase in TVFA and acetate concentrations, as these taxa facilitate more efficient carbohydrate fermentation (Wang et al., 2022; Aguerre et al., 2016). The significant elevation in MCP levels in the T14 group suggests that the optimized nitrogen-to-energy ratio favored the proliferation of nitrogen-utilizing bacteria, enhancing the conversion of ammonia nitrogen into bacterial biomass (Zhu et al., 2020). These findings indicate that dietary protein levels modulate the rumen’s metabolic output by restructuring the microbial ecosystem, specifically by promoting taxa that optimize energy harvest and nitrogen assimilation.

### 4.4 Metagenomics of rumen microbiota

The composition and structure of the microorganisms in the rumen are directly linked with ruminants’ ability to utilize different kinds of feed. The increase in protein content was associated with a concurrent increase of Shannon and Simpson indices, indicating a promotion of rumen microbial diversity. This finding aligned with the results reported in Holstein bulls by Costa-Roura et al. (2020). The current study demonstrated that Firmicutes the main phylum, rather than Bacteroidetes and Proteobacteria, which is consistent with a previous study conducted in Chahaer lambs (Yang et al., 2016). Research has shown that with age, the rumen microbiota of ruminants undergoes significant changes. Numerous studies have confirmed that Firmicutes contain some pathogenic bacteria, which to some extent reflect the dysbiosis or instability of the gastrointestinal microbiota community structure (Byndloss and Baumler, 2018). Bradley and Pollard (2016) demonstrated an association between Firmicutes and inflammation and metabolic syndrome. In this study, we observed that the T14 group exhibits a strong anti-inflammatory effect, suggesting that a high-protein diet can enhance digestion and absorption in goats. We speculate that a low-protein diet can cause inflammation in the body, which in turn affects changes in the abundance of gut microbiota. Another reason is that Firmicutes is associated with the breakdown of proteins (Wang et al., 2022), which is due to the presence of urease in Proteus, which can quickly break down urea and effectively utilize proteins in goats. Liu et al. (2022) reported that Proteobacteria are significantly involved in the degradation of cellulose, hemicellulose, starch, and oligosaccharides, suggesting that goats in group T14 exhibit enhanced degradability of fibrous materials.

At the genus level, the prevalence of *Salmonella* and *Pseudomonas* was lower in group T14 compared to group T8, while *Burkholderia* and *Bacillus* were more abundant. *Salmonella* plays a significant role as a pathogenic factor in gastrointestinal diseases within the US goat industry (Hempstead et al., 2022). *Pseudomonas* is frequently linked to sporadic mastitis in goats (Leitner and Krifucks, 2007). A study by Yusuf et al. (2018) found that the population of *Pseudomonas* in goat meat decreased when the diet was supplemented with 1.5% *Andrographis paniculata* leaves. On the other hand, *Bacilli*, functioning as a probiotic, can directly enhance host health through the production of diverse bioactive substances, regulation of the immune system, and influence on nutrient absorption (Todorov et al., 2022). da Silva et al. (2016) documented that continuous ruminal administration of *Burkholderia* provided protection against poisoning by *Amorimia. Septentrionalis* in goats. In summary, our findings suggest that increasing dietary protein from 8 to 14% leads to improvements in gastrointestinal health by decreasing the relative abundance of pathogenic bacteria and enhancing the relative abundance of beneficial bacteria. This ultimately enhances the digestion and absorption of nutrients, including fiber and protein.

Surprisingly, at KEGG level 3, there was a notable increase in the relative abundance of rumen microbiota genes associated with amino acid metabolism in group T8 compared to group T14. The observed phenomenon is hypothesized to be a result of the compensatory increase that occurs when the protein content of group T8 is in sufficient to support normal microbial growth. This suggests that the rumen microbiome exhibits adaptability under varying diet conditions, despite the differences in rumen microbiome composition between sheep with high and low residual feed intake (Zhang et al., 2021).

### 4.5 Serum metabolomics analysis

The results of the serum metabolomics analysis indicated a significantly higher concentration of substances with anti-inflammatory and anti-oxidative properties, such as genistein and 5-hydroxyindoleacetic acid, in group T14 compared to group T8. Cong et al. (2022) noted that the inclusion of genistein led to a decrease in miR-21 expression by inhibiting NF-kB, consequently leading to a reduction in the inflammatory response. Moreover, Liu et al. (2023) documented that genistein has the potential to reinstate the functions of anti-oxidases and reduce the expressions of NOD-like receptor pyrin domain-containing 3 inflammasome components, thereby alleviating inflammation. Furthermore, Yang et al. (2022) noted that 5-hydroxyindoleacetic acid has been identified as a new ligand for the G protein-coupled receptor (GPR35). This ligand has the ability to enhance neutrophil recruitment to inflammatory sites, facilitating the clearance of pathogenic bacteria through the activation of GPR35. Valente et al. (2021) asserted that the swift breakdown of serotonin in tissues to 5-hydroxyindoleacetic acid could impact the cycling of glucose and insulin, potentially leading to changes in the metabolism of developing cattle. In the current study, the concentration of serum *γ*-glutamylcysteine and L-Cysteine was found to be lower in the T14 group compared to the T8 group. Gamma-glutamylcysteine serves as the precursor for the synthesis of glutathione, which aids in the body’s elimination of detrimental oxygen-free radicals and alleviation of various inflammatory injuries (Braidy et al., 2019). The reduction in levels of γ-glutamylcysteine and L-Cysteine may be attributed to their utilization in the biosynthesis of glutathione. The present study observed enrichment of the glutathione metabolism pathway (Figure 2f). Furthermore, our findings indicated a negative correlation between the relative abundance of *Pseudomonas* and the anti-inflammatory metabolite genistein, while a positive correlation was observed between *Burkholderia* and *Bacillus* with the same metabolite (Supplementary Figure S2). Therefore, it is recommended that increasing the dietary protein level from 8 to 14% enhances the body’s anti-inflammatory and antioxidant capacity, thereby improving production performance. The serum concentration of sulfamethoxazole in group T8 was found to be lower compared to that in group T14. This observation suggests that the goat in group T14 may have possessed a more robust metabolic system, leading to a faster elimination of sulfamethoxazole.

Arginine is recognized as one of the most important amino acids in cells, as it acts as a precursor for the synthesis of various compounds such as nitric oxide, urea, polyamines, proline, glutamate, and creatine (Sawano et al., 2019). Additionally, the interconversion of arginine and ornithine in the body may account for the observed changes in ornithine and imino argininein the context of this study. Furthermore, the enrichment of the arginine and proline metabolism pathway was observed in group T14 compared to group T8. In the body, most of the energy is provided by the crucial metabolic pathways called tricarboxylic acid (TCA) cycle (Liu et al., 2023). Citric acid serves as a primary intermediate in the TCA, and plays a crucial role in mediating glycolysis and gluconeogenesis (Zhao et al., 2020). The rise in gluconic acid levels, as a derivative of glucose, indicates an increase in the availability of glucose. According to Michiels et al. (2023), the inclusion of 9 g/kg of gluconic acid in the diet resulted in improved performance of weaned piglets. The current investigation revealed a significant increase in the levels of citric acid and gluconic acid in group T14 compared to group T8. This finding suggests a potential enhancement in energy metabolism, which could result in increased energy availability for the growth of the T14 group goat (Figure 3). This further elucidates the reasons behind the superior production performance of group T14 goats compared to group T8.

## 5 Conclusion

In order to seek the optimal crude protein requirement for growing Chuannan black goats, 50 goats were selected, and conducted growth, digestion, and metabolism experiments. The results showed that with the increase in dietary CP, there was a decrease in the relative abundance of *Pseudomonas* and *Salmonella*, and an increase in the relative abundance of *Burkholderia* and *Bacillus,* leading to an increase in total VFA, acetate, propionate, and isobutyrate content. The digestibility of DM, CP, and NDF increased, and nitrogen deposition also rose. Metabolomics analysis shows that increasing protein levels can upregulate antioxidant substances such as genistein and 5-hydroxyindoleacetic acid, as well as the final product of the TCA cycle, citric acid. This upregulation leads to the enrichment of arginine biosynthesis, TCA cycle, and glutathione metabolism pathways, thereby promoting goat growth performance. The quadratic regression model indicated that the optimal dietary crude protein level for the growth of Chuannan black goats was determined to be 14%, as determined by the ADG.

## Data Availability

The raw metagenomic data generated in this study have been deposited in the NCBI Sequence Read Archive (SRA) under accession number PRJNA1393407. Other data supporting the findings of this study are included within the article or its Supplementary material.
