# Supplementary material for: The optimal dietary crude protein level improves goat production performance by enhancing the body’s antioxidant function and energy metabolism
Source: Front Microbiol. 2026 Feb 6;17:1734810. doi: 10.3389/fmicb.2026.1734810 (PMC12920459; doi:10.3389/fmicb.2026.1734810)
Supplement: Supplementary file 1 [file Supplementary_file_1.docx]

Table S1 The composition and relative abundance of rumen bacterial communities of group T8 and T14 at the phylum and genus level (%)

| Item | T8 | T14 | SEM | *P*-value |
| --- | --- | --- | --- | --- |
| Phylum level |  |  |  |  |
| Firmicutes | 70.70 | 60.12 | 0.01 | 0.34 |
| Bacteroidetes | 19.48 | 19.58 | 0.04 | 0.87 |
| Actinobacteria | 5.39 | 7.22 | 0.01 | 0.34 |
| Proteobacteria | 2.90 | 8.59 | 0.15 | 0.02 |
| Others | 1.54 | 4.50 | 0.01 | 0.12 |
| Genus level |  |  |  |  |
| *Pseudomonas* | 43.19 | 25.82 | 0.09 | 0.02 |
| *Xanthomonas* | 13.31 | 17.64 | 0.07 | 0.34 |
| *Prevotella* | 15.61 | 13.55 | 0.06 | 1.00 |
| *Streptomyces* | 3.52 | 5.49 | 0.03 | 0.20 |
| *Salmonella* | 5.19 | 1.57 | 0.01 | 0.02 |
| *Bacteroides* | 1.34 | 1.89 | 0.01 | 0.15 |
| *Escherichia* | 1.78 | 1.42 | 0.01 | 0.52 |
| *Burkholderia* | 0.54 | 1.77 | 0.01 | 0.00 |
| *Bacillus* | 0.44 | 1.32 | 0.01 | 0.02 |
| *Bifidobacterium* | 1.18 | 0.16 | 0.00 | 0.42 |
| Others | 13.90 | 29.38 | 0.09 | 0.20 |

Table S2 CAZy annotation map of gene number (Log10 transformed) of group T8 and T14

| Item | Description | T8 | T14 | SEM | *P*-value |
| --- | --- | --- | --- | --- | --- |
| GH | Glycoside hydrolases | 6.28 | 6.23 | 5.62 | 0.01 |
| GT | Glycosyl transferases | 6.06 | 5.92 | 5.46 | 0.02 |
| CBM | Carbohydrate-binding modules | 5.83 | 5.84 | 5.15 | 0.01 |
| AAs | Auxiliary activities | 3.77 | 3.77 | 3.16 | 0.37 |
| PL | Polysaccharide lyases | 4.82 | 4.82 | 4.54 | 0.12 |
| CE | Carbohydrate esterases | 5.34 | 5.17 | 4.77 | 0.04 |

Table S3 Predicted functions at level 3 of the rumen bacterial microbiota of goats of group T8 and T14

| NAME | KO | EC | T8 | T14 | SEM | *P*-value |
| --- | --- | --- | --- | --- | --- | --- |
| **^1^Amino acid metabolism**  **^2^**Alanine, aspartate and  glutamate metabolism (5)  Glycine, serine and  threonine metabolism (5)  Cysteine and methionine  metabolism (5)  Valine, leucine and  isoleucine degradation (3)  Valine, leucine and  isoleucine biosynthesis (1)  Lysine biosynthesis (4)  Lysine degradation (1)  Arginine biosynthesis (4)  Arginine and proline  metabolism (2)  Histidine metabolism (1)  Tyrosine metabolism (2)  Phenylalanine metabolism (2)  Tryptophan metabolism (1)  Phenylalanine, tyrosine and  tryptophan biosynthesis (4) | K00812 | EC[2.6.1.1](https://www.kegg.jp/entry/1.8.1.4) | 3.57 | 3.14 | 3.22 | 0.01 |
|  | K00817 | EC[2.6.1.9](https://www.kegg.jp/entry/1.8.1.4) | 3.46 | 3.00 | 3.06 | 0.01 |
|  | K01928 | EC[6.3.2.13](https://www.kegg.jp/entry/1.8.1.4) | 2.86 | 2.45 | 2.49 | 0.01 |
|  | K00262 | EC[1.4.1.4](https://www.kegg.jp/entry/1.8.1.4) | 3.25 | 2.83 | 2.90 | 0.01 |
|  | K01915 | EC[6.3.1.2](https://www.kegg.jp/entry/1.8.1.4) | 3.22 | 2.89 | 2.82 | 0.02 |
|  | K00928 | EC[2.7.2.4](https://www.kegg.jp/entry/1.8.1.4) | 3.12 | 2.76 | 2.72 | 0.02 |
|  | K12524 | EC[2.7.2.4](https://www.kegg.jp/entry/1.8.1.4) | 3.10 | 2.75 | 2.71 | 0.01 |
|  | K01696 | EC[4.2.1.20](https://www.kegg.jp/entry/1.8.1.4) | 3.10 | 2.68 | 2.74 | 0.03 |
|  | K00382 | EC[1.8.1.4](https://www.kegg.jp/entry/1.8.1.4) | 3.07 | 2.56 | 2.74 | 0.01 |
|  | K00133 | EC[1.2.1.11](https://www.kegg.jp/entry/1.8.1.4) | 3.04 | 2.65 | 2.70 | 0.06 |
|  | K01955 | EC[6.3.5.5](https://www.kegg.jp/entry/1.8.1.4) | 3.00 | 2.69 | 2.62 | 0.05 |
|  | K00826 | EC[2.6.1.4](https://www.kegg.jp/entry/1.8.1.4)2 | 2.93 | 2.58 | 2.46 | 0.10 |
|  | K00800 | EC[2.5.1.19](https://www.kegg.jp/entry/1.8.1.4) | 2.92 | 2.54 | 2.63 | 0.02 |
|  | K00266 | EC[1.4.1.13](https://www.kegg.jp/entry/1.8.1.4) | 2.86 | 2.48 | 2.50 | 0.01 |
|  | K00491 | EC[1.14.14.4](https://www.kegg.jp/entry/1.8.1.4)7 | 2.88 | 2.54 | 2.56 | 0.02 |
|  | K01847 | EC[5.4.99.2](https://www.kegg.jp/entry/1.8.1.4) | 2.88 | 2.48 | 2.45 | 0.01 |
| **^1^Metabolism of other amino acids**  Taurine and hypotaurine  metabolism (1)  Cyanoamino acid metabolism (1)  D-Amino acid metabolism (1) | K13788 | EC[2.3.1.8](https://www.kegg.jp/entry/1.8.1.4) | 2.88 | 2.42 | 2.58 | 0.11 |
|  | K01924 | EC[6.3.2.8](https://www.kegg.jp/entry/1.8.1.4) | 2.86 | 2.50 | 2.55 | 0.06 |
|  | K01921 | EC[6.3.2.4](https://www.kegg.jp/entry/1.8.1.4) | 2.89 | 2.51 | 2.58 | 0.03 |
|  | K05349 | EC[3.2.1.21](https://www.kegg.jp/entry/1.8.1.4) | 3.38 | 2.92 | 3.01 | 0.01 |

**^1^**Representing the function of KO number comparison in KEGG at level 3. **^2^**Representing the number of genes involved in the pathway.

Table S4 Significantly different serum metabolites of T14 vs. T8 group

| Metabolite | RT(s) | FC | *P*-value | VIP | Trend |
| --- | --- | --- | --- | --- | --- |
| Genistein | 800.8 | 17.97 | 0.00 | 2.67 | Up |
| 5-Hydroxyindoleacetic acid | 369.3 | 3.43 | 0.00 | 2.26 | Up |
| m-Cresol | 786.3 | 3.37 | 0.01 | 2.23 | Up |
| Citric acid | 78.5 | 3.01 | 0.04 | 1.22 | Up |
| 2-Heptanone | 190.2 | 2.47 | 0.02 | 1.73 | Up |
| L-Rhamnofuranose | 243.3 | 2.39 | 0.00 | 2.31 | Up |
| Deoxycytidine | 227.7 | 2.39 | 0.04 | 2.03 | Up |
| Adipate semialdehyde | 574.0 | 2.36 | 0.02 | 1.81 | Up |
| L-4-Hydroxyphenylglycine | 153.8 | 2.06 | 0.04 | 1.60 | Up |
| cis-4-Hydroxy-D-proline | 816.5 | 1.93 | 0.04 | 1.77 | Up |
| Ornithine | 80.0 | 1.93 | 0.04 | 1.86 | Up |
| Saccharopine | 101.1 | 1.78 | 0.01 | 1.87 | Up |
| Gluconic acid | 85.6 | 1.72 | 0.01 | 1.77 | Up |
| 8-Hydroxyquinoline | 444.2 | 1.56 | 0.02 | 2.05 | Up |
| Acetylcholine chloride | 72.7 | 0.64 | 0.03 | 2.07 | Down |
| L-2-Hydroxyglutaric acid | 177.4 | 0.62 | 0.01 | 1.88 | Down |
| 1-Methylhistidine | 106.0 | 0.60 | 0.03 | 1.36 | Down |
| trans-1,2-Cyclohexanediol | 837.6 | 0.58 | 0.04 | 1.60 | Down |
| gamma-Glutamylcysteine | 92.5 | 0.57 | 0.00 | 1.67 | Down |
| Adenosine5'-phosphate disodium | 739.9 | 0.56 | 0.03 | 1.94 | Down |
| L-Cysteine | 77.9 | 0.54 | 0.04 | 1.62 | Down |
| Undecanoic acid | 784.7 | 0.51 | 0.03 | 1.64 | Down |
| L-Isoleucine | 101.9 | 0.50 | 0.03 | 1.58 | Down |
| Iminoarginine | 266.7 | 0.36 | 0.04 | 1.85 | Down |
| Capric acid | 744.1 | 0.35 | 0.00 | 1.94 | Down |
| 9,10-DHOME | 682.8 | 0.35 | 0.02 | 1.46 | Down |
| Phenyl acetate | 833.1 | 0.33 | 0.04 | 1.58 | Down |
| 11,14,17-Eicosatrienoic acid | 773.0 | 0.33 | 0.00 | 1.95 | Down |
| L-Histidine | 81.1 | 0.30 | 0.00 | 2.49 | Down |
| (S)-1-Phenylethanol | 699.3 | 0.29 | 0.03 | 1.55 | Down |
| dGMP | 835.1 | 0.28 | 0.00 | 1.91 | Down |
| 6-beta-Hydroxytestosterone | 768.2 | 0.23 | 0.00 | 1.95 | Down |
| Dehypoxanthine futalosine | 90.6 | 0.11 | 0.04 | 1.93 | Down |
| Sulfamethoxazole | 118.0 | 0.02 | 0.00 | 2.54 | Down |
| LysoPA(16_0_0_0) | 769.1 | 0.01 | 0.00 | 1.88 | Down |

RT= retention time; VIP=variable importance in the projection; FC= fold change.

Table S5 Results of different metabolic pathway of T14 vs. T8 group

| Pathway Name | Total |  | Hits^1^ | *P*^2^ | -log10(p) | Impact^3^ |
| --- | --- | --- | --- | --- | --- | --- |
| Histidine metabolism | 16 |  | 2 | 0.10 | 0.99 | 0.22 |
| Arginine and proline metabolism | 38 |  | 2 | 0.02 | 1.61 | 0.11 |
| Arginine biosynthesis | 14 |  | 1 | 0.08 | 1.05 | 0.06 |
| Citrate cycle (TCA cycle) | 20 |  | 1 |  |  |  |
| Glutathione metabolism | 28 |  | 3 | 0.00 | 2.65 | 0.03 |

^1^Hits is the number of metabolites with significant differences in a pathway.

^2^Calculate and correct the *P*-value based on pathway enrichment analysis.

^3^Impact represents the impact value in path topology analysis.

**
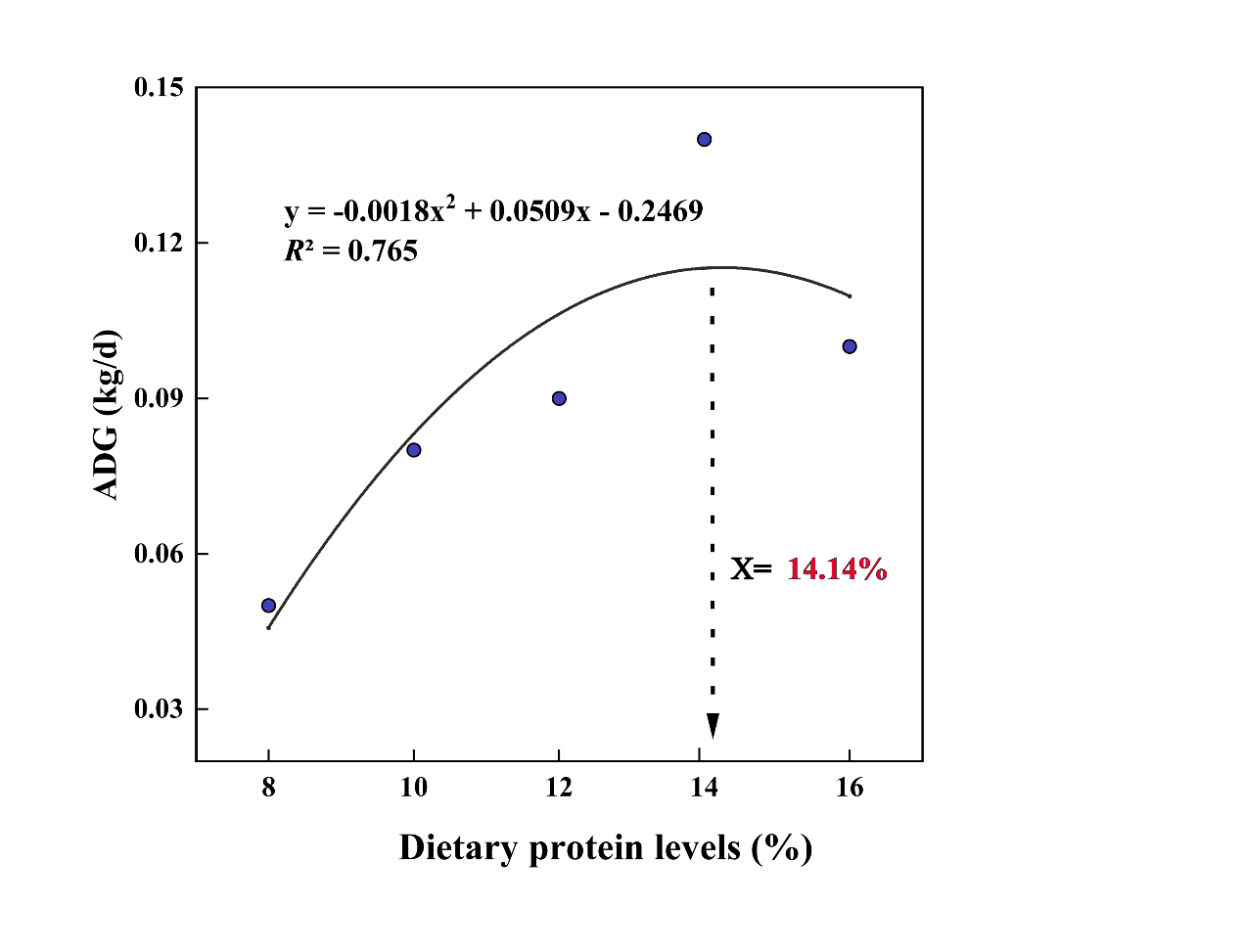
**

Figure. S1. Analysis of the optimal crude protein level in Jintang black goats using a quadratic regression model based on average daily gain


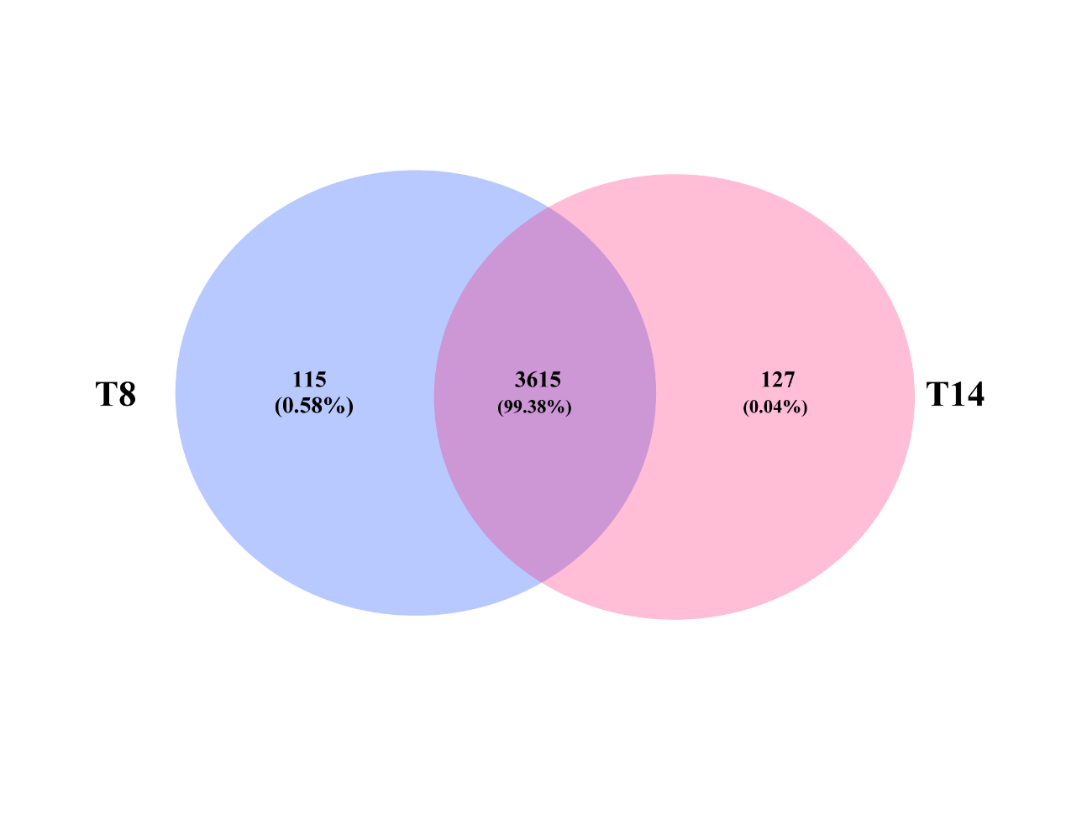


Figure S2. OTUs Venn diagram of group T8 and T14. T8 = Dietary crude protein levels was 8% group; T14 = Dietary crude protein levels was 14% group.


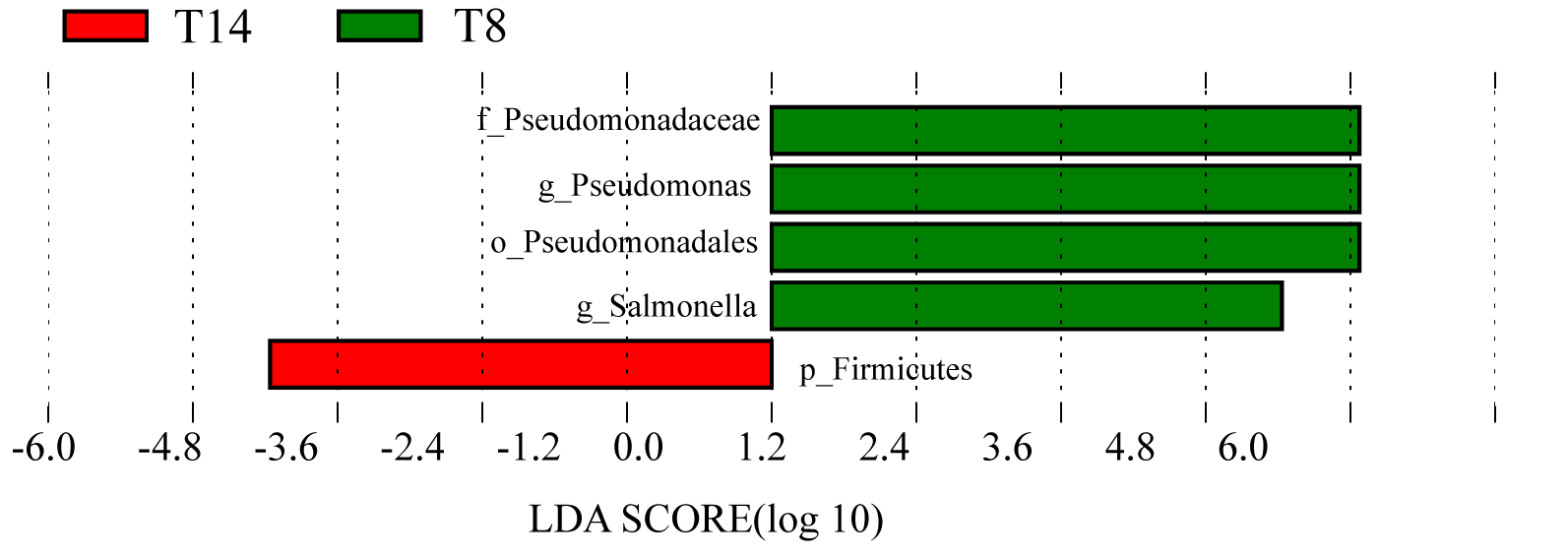


Figure. S3. The linear discriminant analysis effect size (LEfSe) analysis of differential ruminal microorganisms in T8 and T14. T8 = Dietary crude protein levels was 8% group; T14 = Dietary crude protein levels was 14% group.


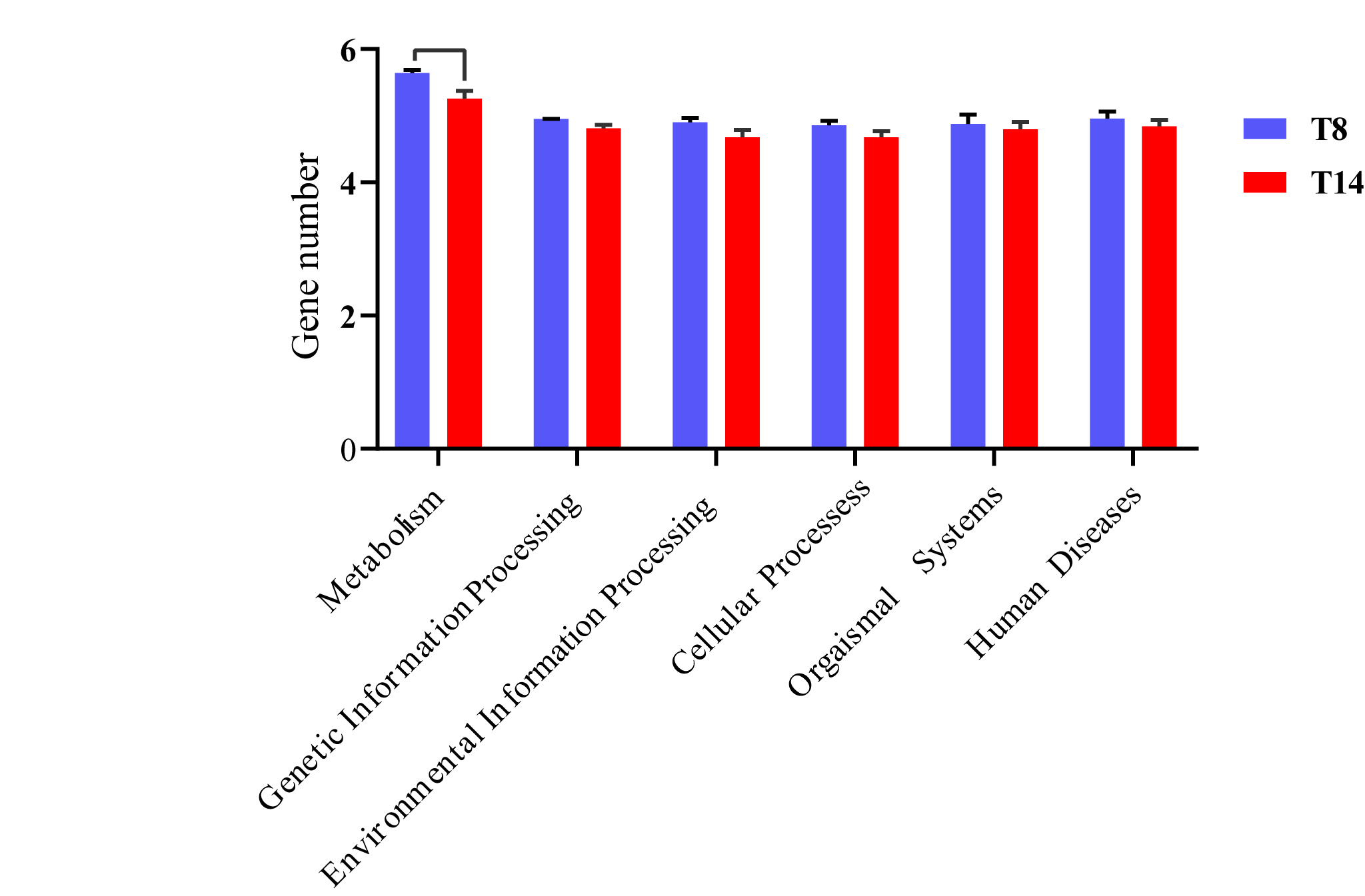


Figure S4. The gene number of different functional taxa at level 1 of the group T8 and T14, annotated based on the KEGG. T8 = Dietary crude protein levels was 8% group; T14 = Dietary crude protein levels was 14% group.





Figure S5. Spearman’s rank correlations between rumen microbiota and serum metabolites; the blue represents negative correlations and red represents positive correlations. The depth of color represents the level of correlation (the higher the correlation, the darker the color); the size of the circle also indicates the level of correlation coefficient, and the higher the correlation coefficient, the larger the circle, **p* < 0.05.
